# Prediction of the Tribological Properties of Polytetrafluoroethylene Composites Based on Experiments and Machine Learning

**DOI:** 10.3390/polym16030356

**Published:** 2024-01-28

**Authors:** Yingnan Yan, Jiliang Du, Shiwei Ren, Mingchao Shao

**Affiliations:** 1 College of Information Engineering, Lanzhou Petrochemical University of Vocational Technology, Lanzhou 730060, China; yanyn11@163.com (Y.Y.); dujiliang@lzpcc.edu.cn (J.D.); 2 Zhuhai Fudan Innovation Institution, Guangdong-Macao In-Depth Cooperation Zone in Hengqin, Zhuhai 519000, China; 3 Key Laboratory of Science and Technology on Wear and Protection of Materials, Lanzhou Institute of Chemical Physics, Chinese Academy of Sciences, Lanzhou 730000, China

**Keywords:** prediction, polytetrafluoroethylene composites, tribology, machine learning, Pearson correlation coefficient

## Abstract

Because of the complex nonlinear relationship between working conditions, the prediction of tribological properties has become a difficult problem in the field of tribology. In this study, we employed three distinct machine learning (ML) models, namely random forest regression (RFR), gradient boosting regression (GBR), and extreme gradient boosting (XGBoost), to predict the tribological properties of polytetrafluoroethylene (PTFE) composites under high-speed and high-temperature conditions. Firstly, PTFE composites were successfully prepared, and tribological properties under different temperature, speed, and load conditions were studied in order to explore wear mechanisms. Then, the investigation focused on establishing correlations between the friction and wear of PTFE composites by testing these parameters through the prediction of the friction coefficient and wear rate. Importantly, the correlation results illustrated that the friction coefficient and wear rate gradually decreased with the increase in speed, which was also proven by the correlation coefficient. In addition, the GBR model could effectively predict the tribological properties of the PTFE composites. Furthermore, an analysis of relative importance revealed that both load and speed exerted a greater influence on the prediction of the friction coefficient and wear rate.

## 1. Introduction

The utilization of PTFE composites has been extensively observed in diverse industrial and scientific research domains owing to their chemical stability, high specific strength, superior process ability, lifetime service at 260 °C, and self-lubrication properties [1,2,3,4,5]. However, the advancement of technology and the increasing demands for applications have created new challenges concerning the development of products that are capable of operating under extreme working conditions due to uncertainties in terms of service characteristics and friction and wear mechanisms [6,7]. Traditionally, the research on tribological properties has mainly relied on trial and error [8,9,10]. The implementation of this approach necessitates extensive experimental research, which not only consumes a substantial amount of time but also fails to provide precise information regarding the service characteristics of PTFE composites. There is currently a lack of methods to predict the tribological properties of PTFE composites, especially under extreme conditions (high temperature or high speed). The assessment of material stability and reliability becomes notably challenging in this scenario. Therefore, it is urgent to find efficient and intelligent methods and technologies to predict friction and wear performance.

To tackle the above problems, researchers have conducted extensive research [11,12]. Among these endeavors, the application of machine learning (ML) technology in the field of materials science has witnessed a gradual increase in recent years, primarily focusing on performance prediction. Cao et al. developed an artificial intelligence graph and neural network method for the structure analysis and performance prediction of a ternary positive crystal through deep learning. The model extracted atomic and chemical bond characteristics from the crystal structure and then predicted the oxidation potential of the crystal and successfully predicted an excellent coating material [13]. Accurately identifying crystal symmetry and enhancing attribute prediction are crucial for overcoming the limitations of current ML algorithms and improving interpretability and generalization. To address the underperformance of conventional ML methods in highly symmetric space groups, Li et al. developed a novel ML model called an isotropic network based on symmetry enhancement [14]. The study conducted by Chen et al. involved the development of four ML models for predicting the maximum adsorption capacity of hydrothermal carbon as well as an exploration of the key factors influencing its adsorption capacity. The results indicated that the gradient boosting decision tree model exhibited exceptional predictive capabilities [15]. The efficacy of ML techniques in addressing intricate nonlinear problems has been well established, demonstrating exceptional performance across various domains and, thus, enabling the application of machine learning in the field of tribology [16,17,18,19]. Zhao et al. successfully predicted the friction coefficient and wear rate of a coating through an ML algorithm of a gradient boosting regression tree. The predictive accuracy for the friction coefficient and wear rate reached 94.6% and 96.3%, respectively [20]. Guo and his team introduced a signal-processing method based on friction noise to predict the tribological properties of polymers over a wide temperature range. Their results indicated that ML methods could effectively predict the friction coefficients of different polymer–metal pairs within a broad temperature domain [21]. Through the application of ML, a novel research concept can be proposed for predicting the tribological properties of polymer composites. However, the current research primarily focuses on low-speed and low-temperature conditions, with limited investigations conducted on predicting tribological properties under high-speed and high-temperature conditions.

In the present study, an optimal ML model was employed to predict the friction coefficient and wear rate of PTFE composites under extreme working conditions. The visualized Pearson correlation coefficient was employed to unveil the quantitative relationship between the working parameters and tribological properties of PTFE composites. The integration of advanced ML techniques enabled the effective prediction and optimization of the service performance of materials in complex working environments, thereby providing guidance for future applications under extreme operating conditions.

## 2. Materials and Methods

### 2.1. Materials and Preparation

**Figure 1:**
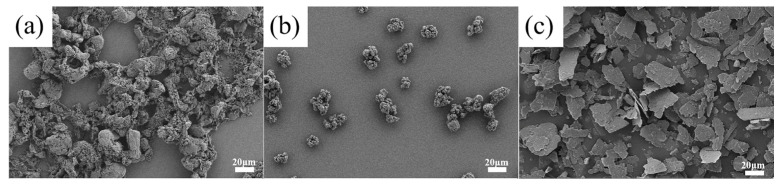
Microstructures of functional fillers: (**a**) PTFE; (**b**) PI; and (**c**) mica.

PTFE powders, with an average diameter of 75 μm, were obtained from Daikin Fluorochemicals Co., Ltd., Changshu, China. Polyimide (PI) powders (YS-20, <75 µm) were procured from Shanghai Synthetic Resin Institute, Shanghai, China. Mica powders were sourced from Shenzhen Haiyang Powder Technology Co., Ltd, Shenzhen, China. The corresponding microstructures of the functional fillers are depicted in Figure 1. The formula of the PTFE composites is shown in Table 1. Mobil Jet Oil II lubricating oil was procured from Beijing AVIC Hangte Lubrication Technology Co., Ltd, Beijing, China. The characteristics of the lubricating oil were as follows: a kinematic viscosity of 5.1 mm^2^/s at 100 °C, with a kinematic viscosity of 27.6 mm^2^/s at 40 °C; specific gravity of 1.00 g/cc; a flash point of 270 °C; a maximum operating temperature of 200 °C as well as 220 °C for a short term; and an operating temperature lower limit of −40 °C.

The specific preparation procedure of the PTFE composites can be consulted for reference [22,23]. Firstly, the fillers were uniformly blended according to a predetermined ratio. Subsequently, a mold with dimensions of Φ145 mm × Φ115 mm was subjected to a pressure of 60 MPa for 4 min. Finally, the PTFE composites were kept warm at 375 °C for 140 min using a sintering furnace. The pressed work-blanks were processed to the specified dimensions in accordance with the test piece requirements.

### 2.2. Tribological Tests

**Figure 2:**
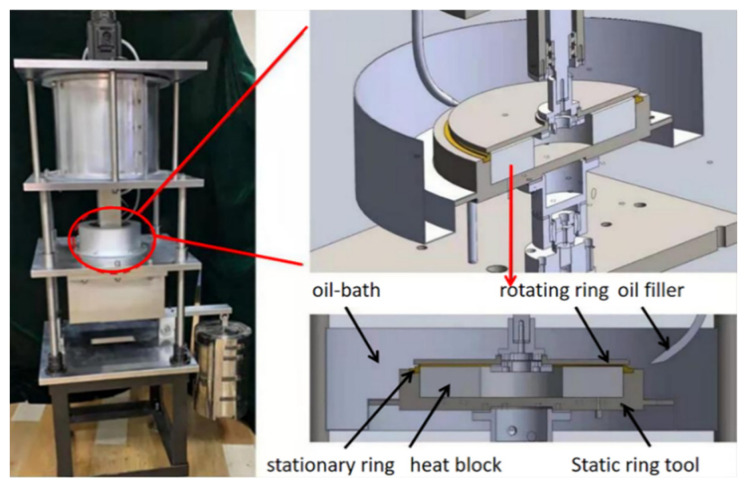
Schematic diagram of the test apparatus.

The tribological performance of the PTFE composites was assessed using a KHS-12000R test apparatus which was prepared by Lanzhou Zhongke Kaihua Technology development Co., Ltd., Lanzhou, China. The structure of the test system is shown in Figure 2. The counterpart was 16Cr3NiWMoVNbE (HRC ≥ 50). The corresponding working conditions and parameters are shown in Table 2. Before the commencement of the experiment, the lubricating oil within the cavity was subjected to heating via a resistance wire. Once the temperature reached the predetermined set point, it was allowed to stabilize for 1 h. A temperature measurement was carried out using a sensor. Each test lasted 10 min and was repeated at least two to three times. The friction coefficient was recorded automatically via a software (Friction and wear testing machine measurement and control system). The friction coefficient of each group was the average of a 6−10 min interval. The wear volume was obtained by multiplying the wear height by the area of the friction sample. The wear height was the average of the values measured at 8 different locations. The wear rate (W, mm^3^/Nm) was calculated using the following formula:

W = ΔV/PL.

Here, ΔV, L, and P represent wear volume (mm^3^), sliding distance (m), and applied load (N), respectively.

### 2.3. Characterizations

The worn morphologies of the PTFE composites were examined by using scanning electron microscopy (SEM, ZEISS Sigma 300, Munich, Germany) to analyze their surface characteristics. The ML algorithms employed were implemented in the Python 3 environment using the open-source Scikit-learn package. To investigate algorithms that were appropriate for modeling intricate friction–wear relationships, random forest regression (RFR), gradient boosting regression (GBR), and extreme gradient boosting (XGBoost) were selected. The original dataset obtained from the friction and wear experiments (see Tables S1 and S2) was randomly divided into a training set and a testing set, ensuring both the generality and accuracy of the models, with a ratio of 7:3. The root mean square error (RMSE) and the coefficient of determination (R^2^) were utilized to illustrate prediction errors and evaluate the performance of the models (which was determined to be outstanding) [24,25,26].

## 3. Results

**Figure 3:**
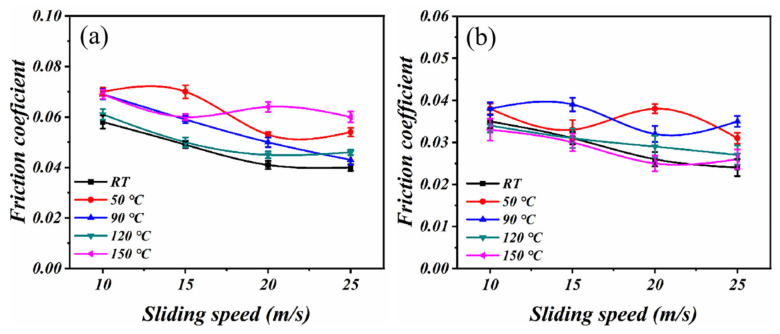
The curves of friction coefficients with speed: (**a**) 0.05 MPa and (**b**) 0.1 MPa.

The relationship between the friction coefficient of the PTFE composites and sliding speed is depicted in Figure 3. The results indicate that the relationship between the friction coefficient and temperature is complex. The friction coefficient generally decreases when the load increases from 0.05 MPa to 0.1 MPa under most working conditions. The reason for this is that it is easier for the lubricant to enter the elastic fluid lubrication area as the load increases. Moreover, an increase in the sliding speed leads to a significant decrease in the friction coefficient. The friction coefficient at this stage is primarily influenced by the internal friction of the lubricating oil.

**Figure 4:**
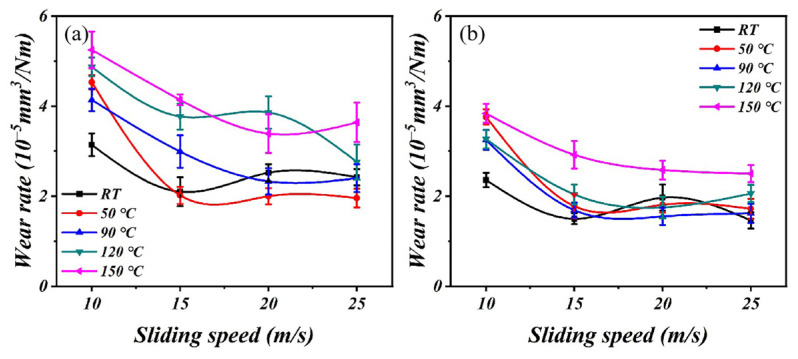
The curves of wear rates with speed: (**a**) 0.05 MPa and (**b**) 0.1 MPa.

The data presented in Figure 4 illustrate a gradual reduction in the wear rate as the speed increases, primarily due to the formation of a continuous and stable lubricating film. The lubricating film acts as a protective layer, effectively preventing direct contact between friction pairs and providing efficient anti-wear protection, thereby significantly reducing the wear rates. The transition from boundary lubrication to elastohydrodynamic lubrication occurs as the load increases, resulting in reduced wear [23]. The influence of temperature on the tribological properties of PTFE composites lubricated with oil is very complicated. When the temperature is low, a continuous lubricating oil film forms between the counterparts, consequently reducing both the friction coefficient and wear rate. The lubricating oil exerts a cooling effect on the friction surface during the process of friction. The rotation of the counterpart facilitates the removal of wear debris from the friction surface by the lubricating oil, thereby minimizing the impact on the tribological performance. The increases in temperature have an important effect on friction and wear performance. On the one hand, they reduce the viscosity of the lubricating oil, thereby diminishing the effectiveness of lubrication. On the other hand, they negate the cooling properties of the lubricating oil and lead to increased wear.

**Figure 5:**
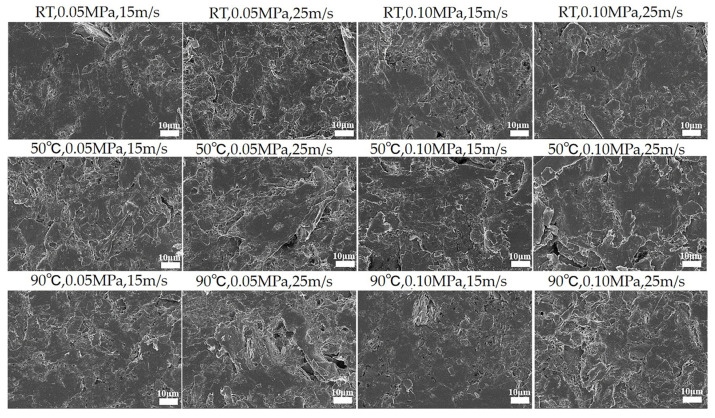
Worn morphologies after subjection to different speeds, temperatures, and loads.

Worn morphologies serve as a direct means of investigating interface changes, thereby providing valuable support for studying the wear mechanisms of PTFE composites. The microscopic worn morphologies of the studied PTFE composites under various operating conditions are depicted in detail in Figure 5 and Figure S1. The aforementioned microscopic morphologies demonstrate that the PTFE composites experienced surface damage and intensified adhesive wear under varying operational conditions, specifically when the speed, temperature, and load increased. The increased severity of working conditions results in a rapid increase in heat accumulation at the friction interface. The temperature of the interface consequently increases, leading to a decrease in the adhesive strength between the filler and matrix that significantly enhances the wear.

**Figure 6:**
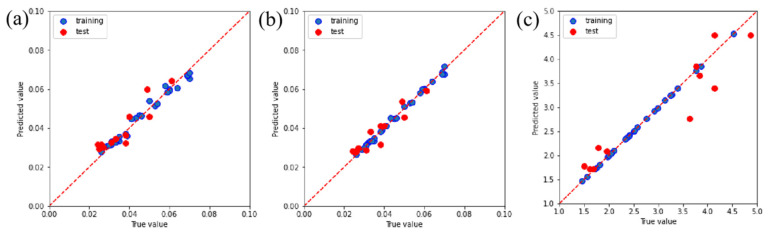
Predictive performance for determining the friction coefficient using the (**a**) RFR, (**b**) GBR, and (**c**) XGBoost models.

**Figure 7:**
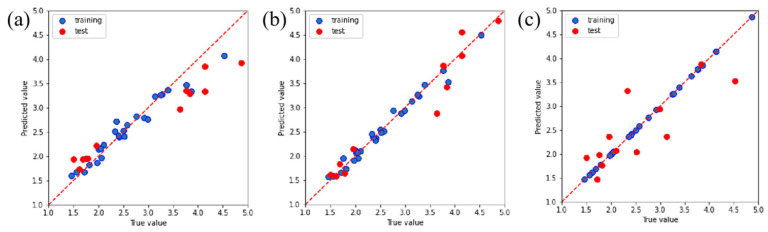
Predictive performance for determining wear rate using the (**a**) RFR, (**b**) GBR, and (**c**) XGBoost models.

The complexity of the friction coefficient and wear rate of PTFE composites under various conditions can be gleaned from Figure 3 and Figure 4. The current wide temperature range (RT to 150 °C) and wide speed range (10 m/s to 25 m/s) complicate the changes in properties that a material exhibits during service. The current research, however, falls short in its predictive capabilities regarding the friction and wear behavior of PTFE composites [27]. Therefore, the RFR, GBR, and XGBoost models were employed for ML to address the aforementioned limitations and acquire predictive capabilities for friction and wear data. The RFR, GBR, and XGBoost models exhibit exceptional functionality and proficiency in handling high-dimensional, non-linear, and intricate data relationships, rendering them well suited for addressing research problems in the field of tribology. After normalizing all experimental data, a model analysis was conducted to ensure the consistency of the data. The predictions of the friction coefficient and wear rate made by the three models are clearly demonstrated in Figure 6 and Figure 7. The results demonstrate the better predictive performance of the GBR model compared to the RFR and XGBoost models, thereby indicating its ability to determine intricate tribological relationships.

**Figure 8:**
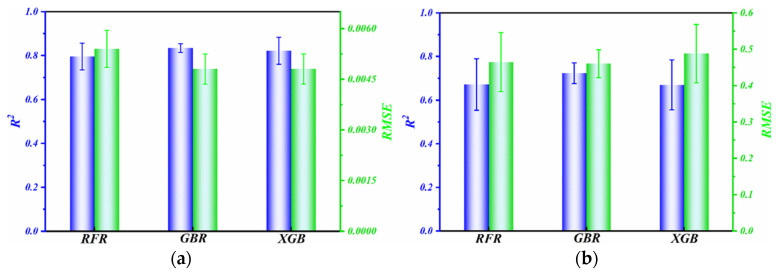
Comparison of the RMSE and R^2^ obtained for the models: (**a**) friction coefficient and (**b**) wear rate.

The specific R^2^ and RMSE values for the RFR, GBR, and XGBoost models are presented in Figure 8. The R^2^ value serves as a metric for assessing the degree of concordance between a model and the corresponding observed data. When interpreting the R^2^ value, values approaching 1 indicate a robust alignment between the model and the data, suggesting that the independent variables can effectively account for most of the variability in the dependent variable. Conversely, if the R^2^ value deviates significantly from 1, this implies that the model has a limited explanatory power. The RMSE measures the extent to which the predictions deviate from the actual outcomes. The significance of the RMSE lies in its capacity to evaluate the efficacy of models, with lower values indicating higher accuracy in outcome prediction. The GBR model exhibited a significantly higher R^2^ value compared to the RFR and XGBoost models, as depicted in Figure 8. Additionally, the GBR model demonstrated a considerably lower RMSE value than the RFR and XGBoost models. The GBR model not only displayed a better fit with the experimental data but also achieved higher prediction accuracy.

**Figure 9:**
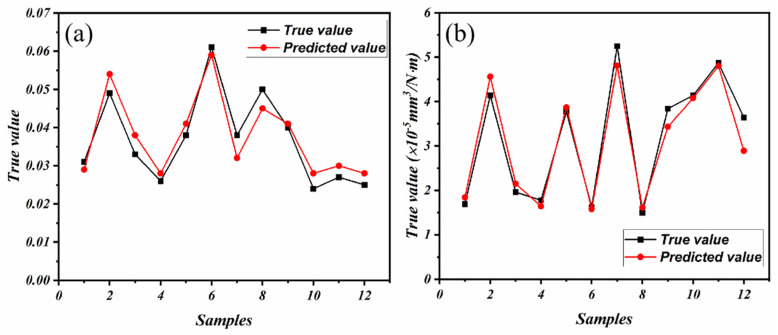
The difference between the predicted and true values of the GBR model: (**a**) friction coefficient and (**b**) wear rate.

The tribological properties of PTFE composites were predicted using the GBR model. The comparison between the predicted values of the friction coefficient and wear rate in Figure 9 demonstrates a high level of agreement with the actual values. The GBR model accurately captured the overall change in the tribological properties, despite some discrepancies between the predicted and real values in certain samples. This numerical result provides support for the screening of the limits of a material’s working conditions.

**Figure 10:**
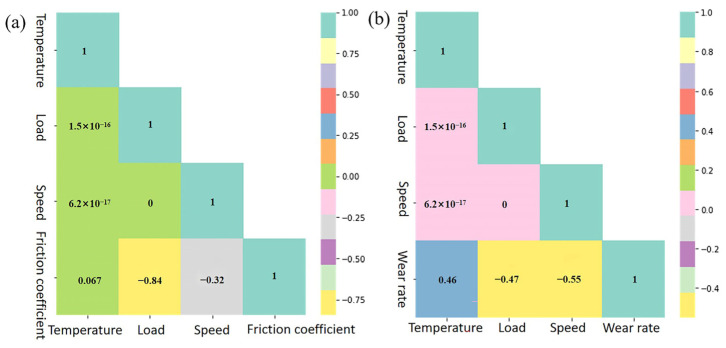
The degrees of influence of different working conditions on tribological properties: (**a**) friction coefficient and (**b**) wear rate.

Currently, analyses of the impact of working conditions on tribological properties are often confounded by extraneous factors. For example, when analyzing the effect of speed on the coefficient of friction, the temperature and load can cause interference. In this study, we employed an ML method to investigate the quantitative impact of a single working condition on the tribological properties while disregarding other concurrent working conditions. The heat map of an ML method is a powerful tool for visualizing the correlation between variables. The correlation coefficient becomes stronger as the color darkens, while it weakens as the color lightens, as illustrated in Figure 10. The correlation coefficients, representing the degree of association between the friction coefficient, wear rate, and various factors, such as temperature, load, and speed, were found to be 0.067, −0.84, −0.32, 0.46, −0.47, and −0.55, respectively. The positive values of these coefficients indicate a positive correlation between the friction coefficient and wear rate and the respective variables, while the negative values suggest a negative correlation. The numerical results obtained from the study provide valuable insights into the influential factors affecting the tribological properties of PTFE composites. Notably, the results demonstrate that the friction coefficient and wear rate are primarily influenced by the load and speed, which emerge as the most significant working conditions. The positive correlation with load suggests that an increase in the load corresponds to an increase in the friction coefficient. Conversely, the negative correlation with velocity implies that higher velocities are associated with lower friction coefficients. This analysis underscores the importance of understanding the individual contributions of working conditions in tribological studies. The intricate interplay between load and velocity elucidates their paramount roles in influencing the performance of PTFE composites. The aforementioned findings contribute to a more nuanced comprehension of the tribological behavior exhibited by materials, thereby facilitating targeted enhancements and optimizations in relevant industrial applications.

## 4. Conclusions

In this communication, the tribological properties of PTFE composites, which provide data support for the predictions of ML models, were investigated through experiments and ML. The corresponding conclusions are as follows:

1. The transition from boundary lubrication to elastohydrodynamic lubrication and the formation of a continuous lubricating film play crucial roles in reducing the friction coefficient and wear rate.
2. The GBR model exhibited better predictive capabilities for both the friction coefficient and wear rate compared to the RFR and XGBoost models.
3. The correlation coefficients between the temperature, load, and speed with the friction coefficient and wear rate were calculated, revealing that the load and speed are the most significant factors influencing the tribological properties of PTFE composites.

The present study offers a valuable direction for the implementation of ML in tribology, thereby presenting a more efficient approach to material selection and design.

## Figures and Tables

**Table 1:** The formula of PTFE composites (mass fraction).

PTFE	PI	Mica
65%	5%	30%

**Table 2:** The corresponding working conditions and parameters.

Operating Conditions	Parameters
Speed (m/s)	10, 15, 20, 25
Temperature (°C)	Room temperature (RT), 50, 90, 120, 150
Load (MPa)	0.05, 0.10

## Data Availability

All data are contained within the supplementary files and this article.

## Supplementary Materials

The following supporting information can be downloaded at https://www.mdpi.com/article/10.3390/polym16030356/s1. Table S1: Friction coefficient of PTFE composites; Table S2: Wear rate of PTFE composites; Figure S1: Worn morphologies under different speeds, temperatures, and loads.
