# Supplementary material for: Prediction of the Tribological Properties of Polytetrafluoroethylene Composites Based on Experiments and Machine Learning
Source: Polymers (Basel). 2024 Jan 28;16(3):356. doi: 10.3390/polym16030356 (PMC10857071; doi:10.3390/polym16030356)

Table S1.

Friction coefficient of PTFE composites.

Temperature (°C)	Load (MPa)	Speed (m/s)	Friction coefficient
25	0.05	10	0.058
25	0.05	15	0.049
25	0.05	20	0.041
25	0.05	25	0.04
25	0.1	10	0.035
25	0.1	15	0.031
25	0.1	20	0.026
25	0.1	25	0.024
50	0.05	10	0.07
50	0.05	15	0.07
50	0.05	20	0.053
50	0.05	25	0.054
50	0.1	10	0.038
50	0.1	15	0.033
50	0.1	20	0.038
50	0.1	25	0.031
90	0.05	10	0.069
90	0.05	15	0.059
90	0.05	20	0.05
90	0.05	25	0.043
90	0.1	10	0.038
90	0.1	15	0.039
90	0.1	20	0.032
90	0.1	25	0.035
120	0.05	10	0.061
120	0.05	15	0.05
120	0.05	20	0.045
120	0.05	25	0.046
120	0.1	10	0.034
120	0.1	15	0.031
120	0.1	20	0.029
120	0.1	25	0.027

150	0.05	10	0.069
150	0.05	15	0.06
150	0.05	20	0.064
150	0.05	25	0.06
150	0.1	10	0.033
150	0.1	15	0.03
150	0.1	20	0.025
150	0.1	25	0.026

Table S2.
Wear rate of PTFE composites.

Temperature (°C)	Load (MPa)	Speed (m/s)	Wear rate ($\times 10^{-5} mm^3/N\cdot m$)
25	0.05	10	3.14
25	0.05	15	2.1
25	0.05	20	2.52
25	0.05	25	2.42
25	0.1	10	2.36
25	0.1	15	1.5
25	0.1	20	1.97
25	0.1	25	1.46
50	0.05	10	4.53
50	0.05	15	2.02
50	0.05	20	2
50	0.05	25	1.96
50	0.1	10	3.76
50	0.1	15	1.78
50	0.1	20	1.81
50	0.1	25	1.72
90	0.05	10	4.14
90	0.05	15	2.99
90	0.05	20	2.33
90	0.05	25	2.4
90	0.1	10	3.25
90	0.1	15	1.69
90	0.1	20	1.55
90	0.1	25	1.62
120	0.05	10	4.87
120	0.05	15	3.77
120	0.05	20	3.86
120	0.05	25	2.76
120	0.1	10	3.27
120	0.1	15	2.04
120	0.1	20	1.75
120	0.1	25	2.06

150	0.05	10	5.25
150	0.05	15	4.14
150	0.05	20	3.39
150	0.05	25	3.64
150	0.1	10	3.84
150	0.1	15	2.92
150	0.1	20	2.58
150	0.1	25	2.5

Figure S1. Worn morphologies under different speeds, temperatures, and loads.

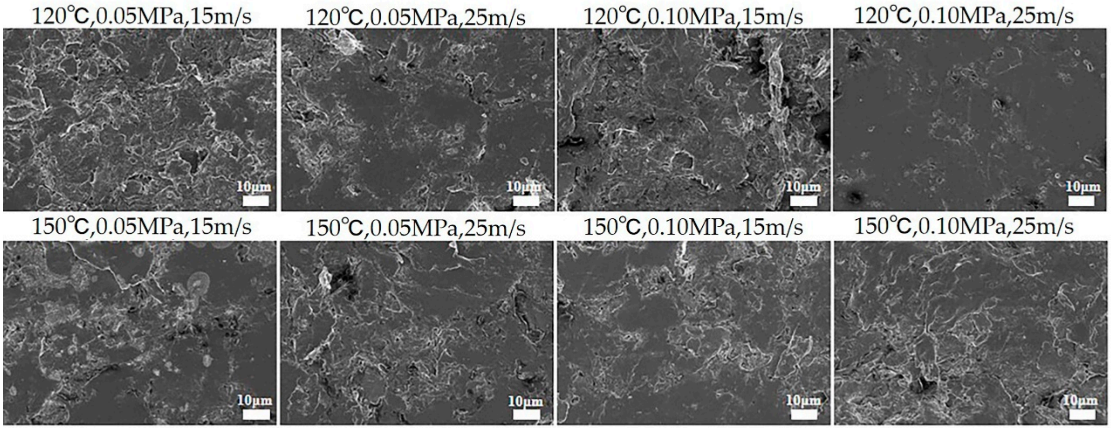

Supplement: Supplementary file 1 [file polymers-16-00356-s001.zip › polymers-2824305-supplementary.pdf]
